# CARD9 Regulation and its Role in Cardiovascular Diseases

**DOI:** 10.7150/ijbs.65979

**Published:** 2022-01-01

**Authors:** Haina Zhang, Yeling Wang, Hongbo Men, Wenqian Zhou, Shanshan Zhou, Quan Liu, Lu Cai

**Affiliations:** 1 Department of Cardiovascular Diseases, First Hospital of Jilin University, Jilin University, Changchun, 130021, China.; 2 Pediatric Research Institute, Department of Pediatrics, University of Louisville, Louisville, KY, 40202, USA.; 3 Departments of Radiation Oncology, Pharmacology and Toxicology, University of Louisville, Louisville, KY, 40202, USA.

**Keywords:** CARD9, innate immunity, inflammation, apoptosis, autophagy, cardiovascular diseases

## Abstract

Caspase recruitment domain-containing protein 9 (CARD9) is an adaptor protein expressed on myeloid cells and located downstream of pattern recognition receptors (PRRs), which transduces signals involved in innate immunity. CARD9 deficiency is associated with increased susceptibility to various fungal diseases. Increasing evidence shows that CARD9 mediates the activation of p38 MAPK, NF-κB, and NLRP3 inflammasome in various CVDs and then promotes the production of proinflammatory cytokines and chemokines, which contribute to cardiac remodeling and cardiac dysfunction in certain cardiovascular diseases (CVDs). Moreover, CARD9-mediated anti-apoptosis and autophagy are implicated in the progression of CVDs. Here, we summarize the structure and function of CARD9 in innate immunity and its various roles in inflammation, apoptosis, and autophagy in the pathogenesis of CVDs. Furthermore, we discuss the potential therapies targeting CARD9 to prevent CVDs and raise some issues for further exploring the role of CARD9 in CVDs.

## Introduction

Despite the development of more advanced therapeutics, cardiovascular diseases (CVDs) remain the leading cause of mortality globally, increasing from 12.1 million in 1990 to 18.6 million in 2019 1. The immune system mainly defends against invading pathogens via a complex interplay between innate and adaptive immunity 2, 3. Immune therapies, particularly immune checkpoint blockade and chimeric antigen receptor T cells, provide a new strategy for treating various cancers 4. In myocardial injury, the immune system evolves into an acute inflammatory response and repair progress 5. Therefore, immune mechanisms underlying CVDs have been explored to elucidate the role of immune responses in cardiac tissue injury and repair, in order to promote the development of efficient therapeutic methods for CVDs.

As a critical adaptor protein, caspase recruitment domain-containing protein 9 (CARD9) is mainly expressed on myeloid cells, downstream of pattern recognition receptors (PRRs) involved in innate and adaptive immunity. Clinically, CARD9 single-nucleotide polymorphisms have been found to be involved in autoimmune diseases, such as Crohn's disease, ulcerative colitis, ankylosing spondylitis, IgA nephropathy, and rheumatoid arthritis 6-9. In fact, CARD9 also plays a role in both infectious and non-infectious pathophysiological processes of heart injuries, including myocarditis, myocardial ischemia reperfusion (I/R) injury, and angiotensin II (Ang II)-induced cardiac remodeling and dysfunction 10-12. Previously, Tian et al. summarized the proinflammatory role of CARD9 in metabolic diseases, including insulin resistance and obesity, which are the risk factors of CVDs, and in heart diseases 13. In contrast, we focus on the various potential roles of CARD9 in CVDs from different perspectives based on recent studies. First, we briefly summarize the structure and function of CARD9 as part of the innate immune response. Then, we describe the activation of CARD9 signaling in CVDs and the role of CARD9 in inflammation-, autophagy-, and apoptosis-mediated myocardial injury and cardiac remodeling in CVDs. Finally, we discuss the clinical therapeutic prospects for CVDs based on the regulation of CARD9 and raise some questions that need to be answered through further investigations on the involvement of CARD9 in CVDs.

## Structure and function of CARD9 in innate immunity

**Figure 1:**
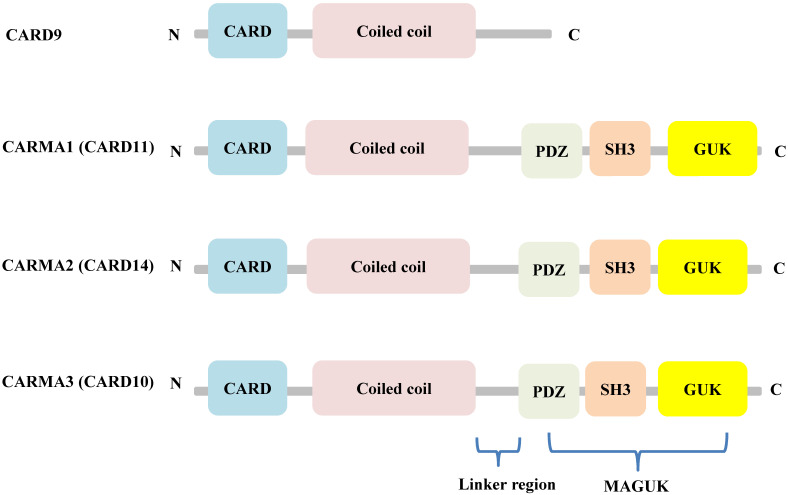
Structural features of the CARMA proteins. CARD9, CARMA1 (CARD11), CARMA2 (CARD14), and CARMA3 (CARD10) consist of homologous N-terminal CARD and C-terminal CCD domain. In addition, CARMA1, CARMA2, and CARMA3 contain the MAGUK domain, including a PDZ, SH3 and GUK domain.

Although CARD9 is highly expressed in myeloid cells, including macrophages, dendritic cells, and neutrophils 14, it is also expressed to some degree in other tissues, including the brain, lung, liver, spleen, and heart 15. Structurally, CARD9 belongs to the scaffold proteins of the caspase recruitment domain (CARD)-containing membrane-associated guanylate kinase (MAGUK) (also called CARMA) family, along with CARMA1 (CARD11), CARMA2 (CARD14), and CARMA3 (CARD10). Unlike the other CARMA family members, CARD9 lacks the C-terminal MAGUK and linker regions that mediate the binding between CARMA proteins and the plasma membrane (Figure 1) 16.

Specifically, CARD9 consists of an amino/N-terminal CARD and a carboxy-terminal coiled-coil domain (CCD) of ~450 amino acids that mediate protein oligomerization 17, 18. Prior to stimulation, CARD9 is held in an autoinhibited state, which is important for maintaining the normal physiological conditions 19-21. Until now, one of the identified structural bases of this autoinhibition is the dependence on an extensive interface between the two domains, CARD and CCD. Once stimulated, CARD9 can be activated via ubiquitination at Lys125 by disrupting the extensive interface or phosphorylation at Thr231 by modulating coiled-coil interactions. Then, the B-cell lymphoma/leukemia 10 (BCL10)-templating filaments of CARD9 are formed, leading to BCL10 activation, which in turn propagates the CARD9/BCL10 signaling cascade 22.

**Figure 2:**
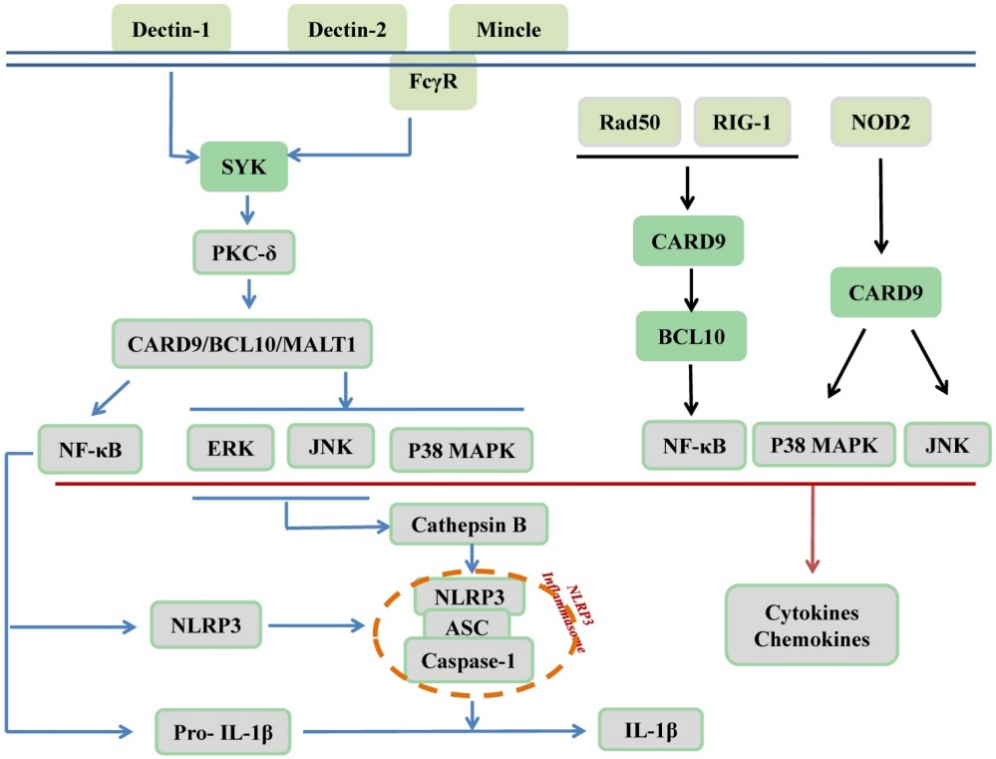
CARD9 signaling in innate immunity. As membrane-bound receptors of PRRs, including Dectin-1, Dectin-2, and Mincle, CLRs recognize β-glucans, α-mannans, and glycolipids, respectively. The ITAM of ITAM-containing receptor (Dectin-1) and ITAM-coupled receptors (Dectin-2, Mincle) can be phosphorylated by the Src family kinase to recruit and activate SYK for further phosphorylation of PKC-δ, subsequently leading to CARD9 phosphorylation. Next, CARD9 recruits BCL10 and MALT1 to form the CBM complex for the activation of MAPKs and NF-κB. The activation of NF-κB can lead to the synthesis of NLRP3 and pro-IL-1β and relayed signals from JNK and ERK pathways induce cathepsin B secretion, thus promoting NLRP3 inflammasome assembly for the production of bioactive IL-1β. In addition, cytosolic PRRs include NOD2, RAD50, and RIG-1. NOD2 recognizes intracellular bacteria and can directly activate CARD9 to activate p38 MAPK and JNK signaling pathways. RAD50 (for viral DNA) and RIG-1 (for viral RNA) can activate CARD9 and further recruit BCL10 to activate the transcription factor NF-κB. Finally, the activation of MAPKs and NF-κB contribute to the production of cytokines and chemokines.

CARD9 is recognized as an adaptor protein and is located downstream of PRRs, which are expressed on innate immune cells. PRRs recognize pathogen-associated molecular patterns (PAMPs) derived from microbes to protect against infectious diseases. As illustrated in Figure 2, C-type lectin receptors (CLRs), as membrane-bound PRRs, including Dectin-1, Dectin-2, and Mincle, are activated by phosphorylation. Src family kinases phosphorylate these three receptors to recruit and activate spleen tyrosine kinase (SYK). Subsequently, SYK phosphorylates protein kinase C (PKC)-δ, which contributes to CARD9 phosphorylation at T231. Finally, the N-terminal of CARD9 recruits BCL10/mucosa-associated lymphoid tissue 1 (MALT1) to form the CARD9/BCL10/MALT1 (CBM) signalosome complex, which activates MAPKs and/or NF-κB to stimulate the production of proinflammatory cytokines, chemokines, and adhesion molecules; however, it remains unclear how and when it specifically activates its downstream MAPKs, NF-κB, or both pathways. Furthermore, the synthesis of NLR family pyrin domain-containing 3 (NLRP3) and pro-IL-1β as the first signal of the NLRP3 inflammasome activation was induced by relayed signals from NF-κB, and cathepsin B secretion, which were induced by relayed signals from JNK and ERK pathways and act as the second signal for production of bioactive IL-1β 14, 23-32. In addition to CLRs, cytosolic PRRs, including nucleotide-binding oligomerization domain-containing protein 2 (NOD2), can directly activate CARD9, thereby promoting p38 MAPK and JNK signaling. Moreover, RAD50 and the nucleic acid sensor retinoic acid inducible gene I (RIG-1) recruit BCL10 to activate NF-κB 17, 33, 34. As revealed by Cao et al., another mechanism responsible for CARD9 activation is ubiquitination by E3 ubiquitin ligase TRIM62 of the K125/Lys125 residue on the CARD9 C-terminal domain to facilitate K27-linked polyubiquitination of CARD9 that subsequently activates NF-κB 35, 36.

**Figure 3:**
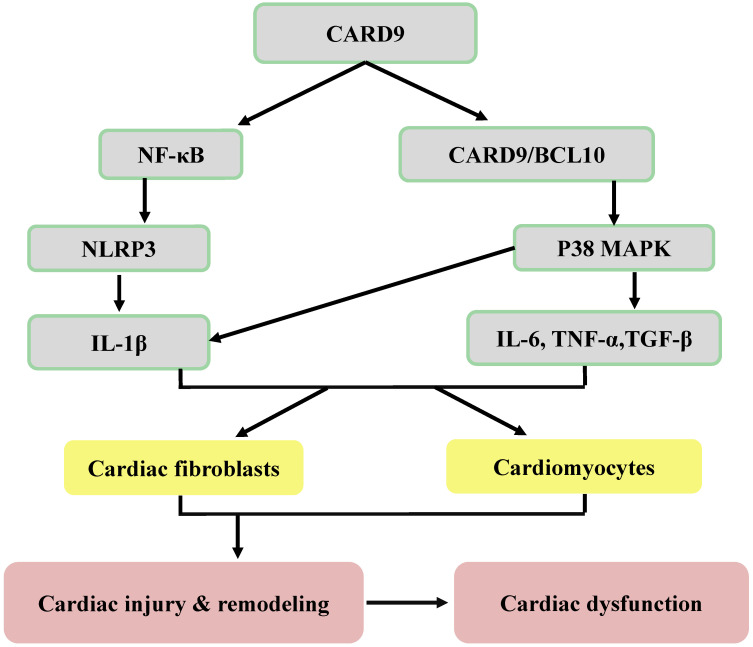
Potential inflammatory mechanisms responsible for CARD9 signaling-induced cardiac dysfunction. Following the infiltration of innate immune cells (macrophages and neutrophils), CARD9 either activates NF-κB or forms a complex with BCL10 to phosphorylate P38 MAPK, leading to an increase of inflammatory cytokines and adhesion molecules. Then these inflammatory cytokines act on both cardiac fibroblasts and cardiomyocytes to mediate cardiac injury and remodeling and can lead to cardiac dysfunction.

**Figure 4:**
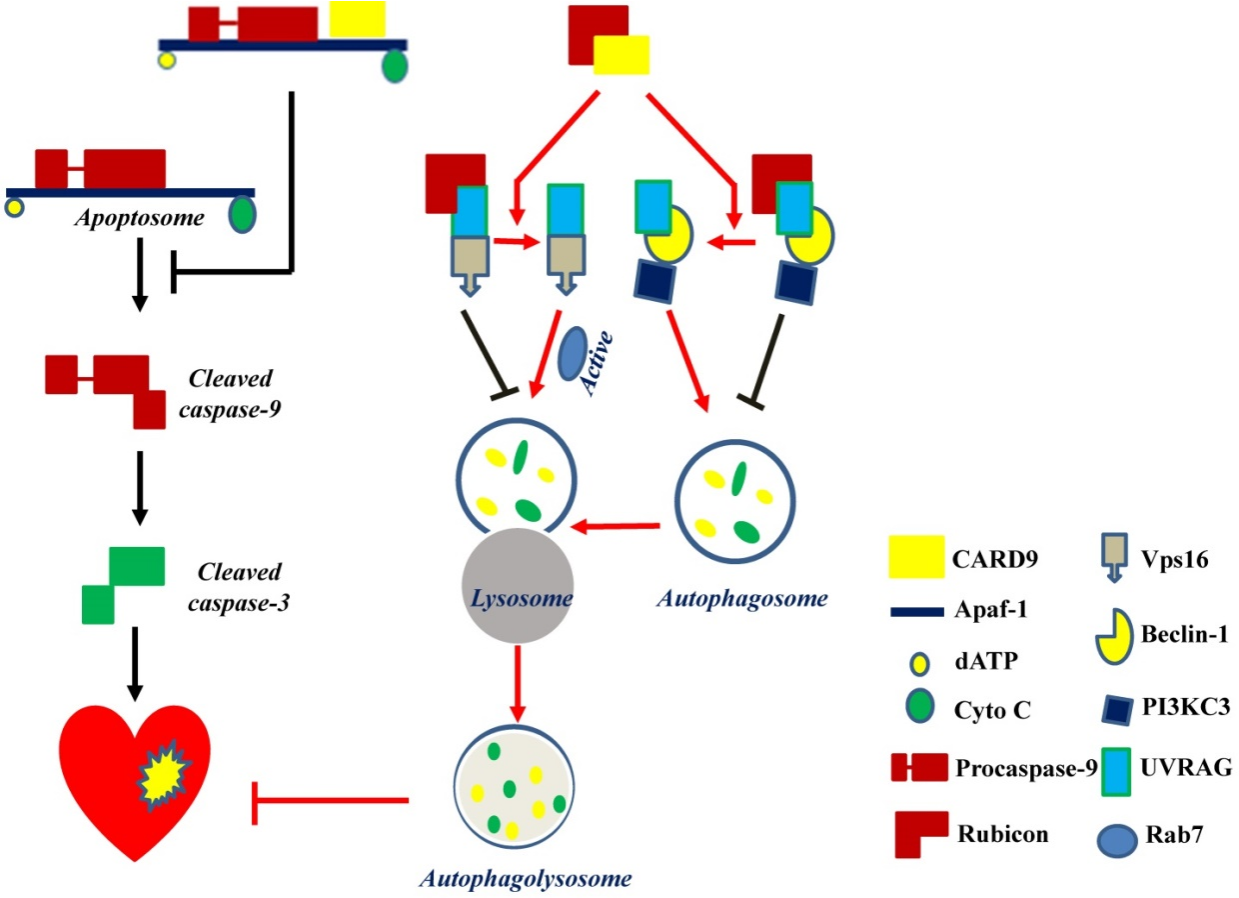
CARD9 protects the heart by inhibiting apoptosis and activating autophagy. CARD9 expressed on cardiomyocytes inhibits apoptosis and promotes autophagy. In CARD9-mediated anti-apoptosis, CARD9 can bind to Apaf-1 to suppress the formation of the apoptosome complex (Apaf-1/procaspase-9), thus inhibiting caspase-9 cleavage-mediated apoptosis. In CARD9-mediated autophagy, CARD9 binds to Rubicon, preventing Rubicon binding to UVRAG for avoiding the formation of a complex with Vps16 or beclin-1/PI3KC3, since Vps16 or beclin-1/PI3KC3 is able to directly or indirectly stimulate autophagosome maturation. Then Rab7, a Rab GTPase superfamily member that promotes the maturation or fusion of the autophagosome with the lysosome, is activated to promote autophagy for the degradation of unnecessary components.

Damage-associated molecular patterns (DAMPs) secreted by damaged and dying host cells can also be sensed by PRRs to promote sterile inflammation through CARD9 signaling. Sterile inflammation is beneficial for tissue repair and regeneration; however, it can also cause inflammatory disorders observed in various diseases, including cancers and CVDs 37. Apart from the role of CARD9 in inflammation, its role in autophagy and apoptosis has been investigated in CVDs (Figures 3, 4). In the following sections, CARD9-mediated pathogenesis in CVDs will be discussed in detail.

## Evidence for the role of CARD9 in CVDs

Sterile and non-sterile infections are known to be involved in the acute and chronic pathological processes of CVDs. In immune inflammation, PAMPs and DAMPs are pathogenic factors in CVDs. As detected in infectious models of acute viral myocarditis and cardiac arteritis 38, 39, PAMPs can induce immune responses that cause acute myocardial injury. In myocarditis induced by coxsackievirus B3 (CVB3), a single-stranded RNA virus, *CARD9*-knockout (KO) mice showed less myocardial inflammation and structural disorganization 10. In addition, *CARD9*-KO mice were also protected from *Candida albicans* water-soluble extract (CAWS)-induced cardiac vasculitis, as evidenced by decreased vascular inflammation score 39.

Meanwhile, DAMPs derived from the sterile CVDs are responsible for advanced cardiac remodeling. S100A8, S100A9 40, 41, and vimentin 42 are recognized as DAMPs in myocardial infarction (MI) and atherosclerosis. In the mouse models of acute MI and myocardial I/R, knockout of *CARD9* contributed to attenuated MI size, indicated by Evans blue and triphenyltetrazolium staining, and decreased neutrophil infiltration, shown by granulocytes-1 immunofluorescence staining 11.

Chronic diseases, such as cardiac hypertrophy, hypertension, and atherosclerosis, account for most CVDs, which are accompanied by inflammatory immune disorders and pathological remodeling in the late stage. In the Ang II-induced cardiac injury model, *CARD9*-KO mice showed less cardiac fibrosis, as demonstrated by less myofibroblast formation 12. In the transverse aortic constriction (TAC)-induced pressure overload mouse model, *CARD9*-KO mice maintained normal cardiac function, and their hearts were not significantly enlarged or fibrotic compared with those of wild-type (WT) mice, which showed pathogenic and functional abnormalities 43 (Table 1). These studies support the pathogenic role of the CARD9-mediated inflammatory pathway in chronic heart diseases.

The upregulation of cardiac CARD9 protein expression along with cardiac fibrosis and dysfunction was recently found in obese mice induced by high fat diet (HFD); however, these effects were blunted in *CARD9*-KO mice 44. In line with this study, we also showed that CARD9 expression and the CARD9/BCL10 complex were increased in HFD-induced obese mice with cardiac hypertrophy 45. These studies suggest the essential role of CARD9 in obesity‑induced cardiac remodeling and dysfunction. However, deletion of hematopoietic *CARD9* did not exhibit a protective effect in atherosclerosis. Instead, hematopoietic *CARD9*-KO showed an increase or no influence on atherosclerotic lesion area and lesion severity in hyperlipidemic 46 and hyperglycaemic mice 47, suggesting that CARD9 plays a different role in different conditions and pathogenic models.

Taken together, the above studies (summarized in Table 1) have demonstrated the important pathogenic roles of CARD9 in various CVDs. Although CARD9 is mainly expressed on myeloid cells, its expression is also detected in endothelial cells and cardiomyocytes in response to different stimuli 48-50. Therefore, the pathogenic role of CARD9 in atherosclerosis may be attributed to different experimental conditions, such as types of cells used (global KO vs. hematopoietic cell KO). Understanding the potential underlying mechanisms can further clarify the specific involvement of CARD9 in CVDs.

## Potential mechanisms underlying the pathogenic effects of CARD9 in CVDs

Inflammation, apoptosis, and autophagy are involved in the initiation and development of CVDs. As a key adaptor of innate immune molecules, CARD9 is involved in these cellular events. In the following sections, we describe the pathogenic mechanisms of CARD9 in CVDs.

### CARD9 and inflammation

CARD9 plays an essential role in cardiac injury, remodeling (hypertrophy and fibrosis), and dysfunction 12, 43-45. During cardiac dysfunction, the CARD9 signaling-mediated production of inflammatory cytokines (IL-6, IL-1β, TNF-α, and TGF-β) and chemokines (MCP-1/CCL2 and CXCL1), which are mainly secreted by macrophages and neutrophils, in the heart has been investigated.

#### p38 MAPK in CARD9-mediated inflammatory pathogenesis

As shown in Figures 2 and 3, the CARD9-mediated activation of p38 MAPK is a major mechanism responsible for the transcription and production of inflammatory cytokines and chemokines, which are involved in the recruitment of immune cells, including macrophages and neutrophils, and in the pathological mechanism of CVDs 51-53. Cardiac structural remodeling, one of the pathological outcomes of CVDs, includes cardiac hypertrophy (predominantly cardiomyocyte hypertrophy) and fibrosis (mainly in the cardiac extracellular matrix). Early remodeling often compensates for the loss of certain cardiomyocytes caused by various pathological stimuli; however, upon the transition to decompensation from compensatory remodeling, cardiac dysfunction occurs, leading to heart failure (HF) or cardiac arrest due to cardiac electrical remodeling. In patients with HF, the levels of inflammatory cytokines, including IL-6, IL-1β, and TNF-α, were increased and correlated with disease severity 54, 55. These cytokines can affect cardiomyocytes and fibroblasts to mediate cardiac hypertrophy and fibrosis. IL-6 induces cardiac fibroblasts to express enhanced fibrosis-related factors in aldosterone-induced cardiac fibrosis *in vitro*, whereas its inhibition can prevent myocardial fibrosis and cardiac hypertrophy *in vivo* 56. In addition, IL-6 infusion in healthy animals directly induces cardiomyocyte hypertrophy and cardiac fibrosis 57; in contrast, the deletion of *IL-6* decreased pressure overload-induced ventricular hypertrophy 58, suggesting a direct pathogenic effect of IL-6 on the heart. Therefore, Tocilizumab, a human anti-IL-6 receptor antibody for the IL-6 blockade, has been clinically applied to prevent atherosclerosis, MI, and even COVID19-related cardiac injury 59-61. For TNF-α, its cardiac-specific overexpression in mice can cause ventricular hypertrophy and cardiac fibrosis, leading to HF 62, whereas *TNF-α*-KO mice showed less cardiac hypertrophy and reparative fibrosis in response to pressure overload 63.

Myeloid cells are crucial in cardiac remodeling 64. In a mouse model of obesity, cardiac CARD9 expression was increased, and the activation of p38 MAPK was augmented 44, 45. As discussed above, CARD9 upregulates cytokines IL-6, IL-1β, and TNF-α. Deletion of *CARD9* downregulates p38 MAPK activity and reduces the levels of these inflammatory cytokines and macrophage infiltration, and improves cardiac hypertrophy, fibrosis, and dysfunction 11, 12, 44, 45. The essential role of p38 MAPK in CARD9-mediated cardiac pathogenesis was defined by the fact that inhibition of p38 MAPK function both *in vitro* and *in vivo* using its inhibitor, SB203580, did not change the expression of CARD9, but it significantly inhibited the production of inflammatory cytokines, including IL-6 and TNF-α, and cardiac pathogenesis 45,65 (Figure 3).

As one of the downstream targets of p38 MAPK, TGF-β has also been shown to contribute to pressure overload-induced cardiac fibrosis by activating Smad2/3 signaling in activated cardiac fibroblasts 66-70. For instance, the inhibition of p38 MAPK significantly downregulated the mRNA levels of TGF-β signaling-related genes, including *Tgfb2* and *BMP4* 69. TGF-β antibody treatment suppressed Smad activation in the interstitium but not in cardiomyocytes 68. Moreover, the inhibition or knockdown of p38 MAPK using its inhibitor PH797804 or small-interfering RNA in isolated right ventricular fibroblasts of mice attenuated TGF-β-induced Smad2/3 phosphorylation and blocked TGF-β-induced fibroblast transdifferentiation 70. Furthermore, the TGF-β signaling pathway was upregulated in the heart of a pressure overload mouse model 68, and in patients with hypertrophic cardiomyopathy 69. Similarly, the deletion of *CARD9* resulted in less Ang II-induced cardiac fibrosis and decreased activation of p38 MAPK and TGF-β expression 12 (Figure 3). Therefore, these studies indicate that CARD9 located upstream of these cytokines could be a potential therapeutic target to protect against cardiac remodeling.

In addition to its involvement in TGF-β-mediated cardiac remodeling, the inhibition of p38 MAPK attenuated acute cardiac injury and improved heart function in chronic CVDs, such as myocardial I/R injury and diabetic cardiomyopathy 71, 72 via different signaling pathways. We found that in obesity-induced cardiac hypertrophy mice, obesity led to increased CARD9 expression, p38 MAPK activation, and the increased expression of the hypertrophy-related genes of *GATA4* and *MEF2c*. Inhibition of p38 MAPK activity then decreased *GATA4* and *MEF2* expression, but CARD9 expression was unchanged. Thus, CARD9 could also be involved in the pathogenesis of cardiac hypertrophy via p38 MAPK-mediated activation of the GATA4 and MEF2 signaling pathways 45, 65.

These results support the notion that the activation of CARD9-mediated p38 MAPK-dependent inflammation could be a vital pathogenic mechanism that leads to cardiac injury and remodeling. Thus, inhibiting p38 MAPK-dependent overactive inflammation could be a potential therapeutic target for CVDs (Figure 3).

#### NF-κB and its downstream key component, NLRP3 inflammasome, in CARD9-mediated pathogenesis

As summarized in Figures 2 and 3, NF-κB is another component of the CARD9-activated downstream inflammatory pathways, in parallel with p38 MAPK 12, 73. Unlike in the immune system, it is inconclusive whether NF-κB activation is regulated by CBM in CVDs, since experiments measuring the signalosome complex are lacking. NF-κB can activate multi-inflammatory cytokines and the NLRP3 (previously known as NACHT, LRR, PYD domain-containing protein 3, and cryopyrin) inflammasome, which is an essential inflammatory pathway component in the pathogenesis of CVDs 74-76. Generally, the activation of NLRP3 triggers a rapid oligomerization of the inactive pro-caspase-1 enzyme and the ASC adaptor protein to form the NLRP3 inflammasome that cleaves precursor pro-IL-1β into the mature and bioactive proinflammatory cytokine IL-1β 77, which is involved in innate immunity 78,79 and CVDs 80,81.

Compared with WT mice, *CARD9*-KO mice showed significant resistance to pressure overload-induced cardiac remodeling and dysfunction and exhibited NF-κB inactivation 43. In an Ang II-induced cardiac remodeling model, *CARD9*-KO mice presented less cardiac fibrosis and macrophage infiltration, no significant activation of NF-κB, and defective expression of IL-1β 12. In another study, oxidized low-density lipoprotein (oxLDL) immune complexes (ICs), which are strong predictors of CVDs 82, induced increased transcription of inflammasome-related genes, including *Nlrp3*, *Il1a*, *and Il1b*, in WT bone marrow (BM)-derived dendritic cells (BMDCs) but not in *NLRP3*-KO BMDCs 83. In addition, *CARD9* deficiency in BMDCs not only decreased the oxLDL ICs-induced expression of inflammasome-related Nlrp3 mRNA and the expression of IL-1β, but also inhibited the nuclear translocation of NF-κB 83. Collectively, CARD9 promotes NF-κB translocation, Nlrp3 transcription, and IL-1β cleavage in BMDCs treated with oxLDL ICs (Figure 3).

Dectin-2 is stimulated by microbial signals through SYK and CARD9, thereby contributing to NF-κB activation 84. Following the systemic administration of CAWS extract to induce cardiac arteritis 39, Miyabe et al. reported the infiltration of inflammatory monocytes (peaked at day 1), monocyte-derived dendritic cells (Mo-DCs; increased at day 7 and continued until day 28 at approximately the same level), and neutrophils (observed at day 1 and peaked at day 28) into the aortic root and coronary arteries. Then, they tracked the transferred WT BM-derived monocytes labeled with green fluorescent protein and found the differentiation of Mo-DCs from inflammatory monocytes 39. After CAWS extract injection, of all inflammatory cytokines, IL-1β was the most highly expressed in the heart on day 28, followed by IL-6 and TNF-α. On the other hand, *IL-1α/β*-KO mice did not develop CAWS-induced arteritis 39, confirming the direct pathogenic role of IL-1β in CAWS-induced arteritis. In addition, it has been reported that the Dectin-2/CARD9/NLRP3 inflammasome signaling in Mo-DCs from the heart is required for IL-1β secretion, based on the following evidence: first, mice with knockout of *Dectin-2*, *CARD9,* or mutant *SYK*, as well as mice with knockout of *Nlrp3* or* Caspase-1*, were completely protected from vasculitis 39. Second, mice with deleted *Dectin-2* or mutant *SYK* showed reduced IL-1β levels 39. Finally, compared with WT BMDCs, NF-κB p65 translocation to the nucleus, a key step in the activation of the NF-κB signaling pathway, was abolished in BMDCs with *Dectin-2* deficiency following CAWS administration 39. Overall, the Dectin-2/SYK/CARD9 pathway mediates NF-κB activation and the NLRP3 inflammasome-induced production of IL-1β in CAWS-induced vascular injury.

To date, only a few studies have investigated the involvement of CARD9 signaling-mediated NLRP3 inflammasome formation in CVDs; however, we can widen our understanding indirectly through other studies. CARD9 upregulates IL-1β production in fungal infection, whereas it negatively regulates the NLRP3 inflammasome-induced IL-1β production in BMDMs in response to *Salmonella* infection. In this study, CARD9 inhibited SYK phosphorylation and subsequently suppressed NLRP3 activation and the recruitment of caspase-8 to the inflammasome, resulting in decreased IL-1β levels 85. Thus, SYK not only acts as an upstream mediator of CARD9 but is also regulated by CARD9 through changing SYK phosphorylation status to form a feedback regulation loop. Therefore, the role of CARD9 in regulating NLRP3-induced CVDs requires further study.

In summary, signals from CVDs can induce the activation of CARD9 signaling following the infiltration of monocytes and neutrophils. Subsequently, p38 MAPK and NF-κB inflammatory pathways are activated. Cardiac injury and remodeling can occur after the release of proinflammatory cytokines, including IL-6, IL-1β, TNF-α, and TGF-β. To date, our understanding of the role of CARD9 signaling-mediated inflammatory responses in infectious or aseptic cardiac diseases is consistent, indicating that CARD9 contributes to proinflammatory effects.

### Apoptosis in CARD9-mediated pathogenesis

Cardiomyocyte apoptosis can induce cardiac remodeling and lead to cardiac dysfunction in various CVDs 86-88, and CARD9 plays a role in this process.

Mitochondria-dependent apoptosis is a prominent form of myocardial cell death after cardiac I/R injury and is characterized by the formation of the apoptosome, a complex comprising the apoptotic protease activating factor-1 (Apaf-1), cytochrome C, dATP, and procaspase-9 89. Reperfusion can trigger respiratory chain uncoupling, leading to ROS production 90. Excessive ROS induces cardiomyocyte apoptosis and leads to cardiac dysfunction. An *in vitro* study reported an increase in CARD9 expression in H9C2 cardiac cells in response to hydrogen peroxide (H_2_O_2_). The knockdown or overexpression of CARD9 increased or inhibited cardiac apoptosis in response to H_2_O_2_, respectively. Meanwhile, compared with WT cardiac I/R mice, *CARD9*-KO mice had a higher number of apoptotic cells in the heart tissue 50. These results indicate the anti-apoptotic role of CARD9. Li et al. subsequently found that CARD9 overexpression in H9C2 cells blocked H_2_O_2_-induced caspase-9 cleavage without affecting cytochrome C release. On the other hand, its downregulation increased the activation of caspase 9 in response to H_2_O_2_. Furthermore, in *CARD9*-KO H9C2 cells, the inhibition of caspase-9 activity reduced H_2_O_2_-induced apoptosis, suggesting that the increased apoptosis by CARD9 is mediated by the activation of caspase-9. Further mechanistic studies showed that the CARD domain of CARD9 contributed to mitochondria-dependent apoptosis, as this effect was disappeared in the mutant CARD9 with a deleted CARD domain 50. Moreover, co-immunoprecipitation assay and immunofluorescence staining revealed that CARD9 binds Apaf-1 to prevent the formation of the Apaf-1/procaspase-9 complex (apoptosome) in response to H_2_O_2_ in H9C2 cells 50. Collectively, in H9C2 cells under oxidative stress, the CARD domain of CARD9 binds to Apaf-1 to disrupt apoptosome formation, thereby suppressing caspase 9 activation and apoptosis (Figure 4).

These findings suggest that CARD9 expressed on cardiomyocytes exhibited an anti-apoptotic role to protect the heart, which is different from its role in inflammation. Due to the limited number of studies on CARD9-mediated apoptosis in CVDs, we expected to find some clues from studies conducted on targets upstream of CARD9 to better explain this difference.

In accordance with the adverse consequence of CARD9-mediated inflammation in the heart, Yan et al. observed improved infarct healing and an attenuated cardiomyocyte apoptotic rate in *Dectin-2*-KO mice at day 3 post-MI 91. They also found that the knockout or inhibition of Dectin-1 improved cardiac function and reduced cardiomyocyte apoptosis in a cardiac I/R mouse model 92. Apart from the different parts of CARD9 signaling, this discrepancy in the effect of CARD9 signaling on apoptosis may be due to the differences in the cell types studied. Particularly, CARD9 expressed on cardiomyocytes showed an anti-apoptotic role 50, whereas its expression on myeloid cells might play a pro-apoptotic role 91, 92. In brief, different cell contexts could have their own regulatory mechanisms of apoptosis. In addition, the former anti-apoptotic mechanism 50 was revealed in an *in vitro* cell study that did not measure cardiac function, and the time point of apoptotic rate measurement was ahead of the latter studies 91, 92. The underlying mechanisms responsible for these different outcomes should be investigated in future studies.

### Autophagy in CARD9-mediated pathogenesis

Efficient flux through the autophagic pathway is essential for cell survival 93, 94. In CVDs, autophagy is a double-edged sword. On the one hand, it is an adaptive response to stress conditions for limiting cardiac damage. On the other hand, excessive autophagy activation could be detrimental for the heart in some stress conditions 95-97.

Myocardial reperfusion is an effective therapy for acute MI. Minimizing blood flow resumption time is critical to treat acute MI and reduce I/R injury. Autophagy in cardiomyocytes is activated by ROS following myocardial I/R injury 98. CARD9 expression was increased in myocardial I/R mouse hearts and in H9C2 and neonatal rat ventricular myocytes in response to hypoxia/reoxygenation (H/R) or H_2_O_2_. The deletion of *CARD9* aggravated cardiac damage and dysfunction and impaired autophagy during myocardial I/R both *in vivo* and *ex vivo*. In contrast, its overexpression in H9C2 cells subjected to the same H_2_O_2_ concentration resulted in increased autophagic flux as indicated by the increased LC3II/I ratio, autophagosome formation, and decreased p62 expression 49. The inhibition of autophagy using 3-methyladenine or Bafilomycin A1 abolished the protective role of CARD9 overexpression in H/R or oxidatively stressed cardiac cells induced by H_2_O_2_, suggesting that the CARD9-mediated cardiac protection from oxidative stress is mediated by autophagy stimulation 49 (Figure 4).

Regarding autophagy, there may be three key steps for the flux: autophagosome formation, maturation by the fusion of autophagosomes with lysosomes, and autophagolysosome formation. The formation of autophagosomes can be stimulated by the formation of a complex comprising UV-irradiation-resistance-associated gene (UVRAG), beclin-1, and the phosphatidylinositol 3-kinase catalytic subunit type 3 (PI3KC3). Meanwhile, the fusion of autophagosomes with lysosomes can be activated by Rab7, a member of the Rab GTPase superfamily that can be activated by forming a complex of UVRAG with the vacuolar protein sorting-associated protein 16 homolog (Vps16) (Figure 4). However, Rubicon can also bind UVRAG to interrupt its interaction with beclin-1/PI3KC3 or Vps16, thus inhibiting autophagosome formation or maturation, respectively 49 (Figure 4). Therefore, Rubicon is a well-recognized negative regulator of autophagy. Based on this notion, Li et al. revealed that CARD9 can competitively bind Rubicon with UVRAG, resulting in the release of UVRAG to form a complex with beclin-1/PI3KC3 or Vps16 and ultimately facilitating autophagosome formation, maturation, and endocytosis 49. Above all, CARD9 expressed in cardiomyocytes interacts with Rubicon to indirectly upregulate autophagy and protect the heart from myocardial I/R-induced cell death and injury 49, as illustrated in Figure 4.

However, in the models of HFD-induced obesity and TAC-induced hypertension *CARD9*-KO alleviated myocardial dysfunction and restored dysfunctional myocardial autophagy accompanied by an increased ratio of LC3BII/I and decreased p62 expression 43, 44. These results are contrary to the protective role of CARD9 described in the previous paragraph. This inconsistency can be due to the use of different models: the former studied an acute injury model 49, whereas the latter investigated models of chronic disease 43, 44. Of note, even in the same model, two studies also reported conflicting results 11, 49: one study revealed that the knockout of *CARD9* exerts a protective effect on myocardial I/R-induced infarct size 11, while another reported that the knockout of *CARD9* resulted in a cardiac dysfunction 49. These conflicting results may be due to the different conditions of the experimental model they used: the protective effects were observed in mice with 45 min occlusion of left anterior descending (LAD) coronary artery, followed by 24 h reperfusion 11 while the detrimental effect was observed in the mice with 30 min LAD occlusion, followed by reperfusion for 12 h 49. In addition, the types of cells expressing CARD9, i.e., cardiomyocytes or innate immune cells, may also contribute to those inconsistent results. To confirm and validate these results, more studies focus on CARD9 (expressed on different cell types) mediated CVDs are needed.

## Therapeutic prospects

Regarding MI therapy, the injection of pluripotent stem cells has been investigated. In a MI model injected with stem cells, *CARD9*-KO mice showed a reduction in early death rate and a decreased formation of granulomas containing macrophages and T cells 99. Coronary artery bypass grafting is an important invasive vein graft strategy for coronary artery disease that aims to restore adequate blood supply to save ischemic hearts due to severe coronary stenosis or vessel occlusion. At present, progressive intimal hyperplasia and atherosclerosis have become the main constraints of long-term outcomes in vein grafts 100. A recent study showed that CARD9 is highly expressed in macrophages infiltrating vein grafts 101. Knockout of CARD9 reduced the necrotic smooth muscle cell-induced macrophage infiltration, NF-κB activation, and the expression of proinflammatory molecules, including IL-6. This subsequently resulted in decreased neointima formation in grafted veins in *CARD9*-KO mice 101. Thus, the inhibition of CARD9 prevented macrophage-mediated acute immune injury in stem cell therapy for MI and could be a target to avoid vein graft remodeling and failure.

For clinical applications, effectiveness and safety are the two main considerations. To date, small molecules targeting posttranslational modifications of CARD9 have not been applied in the treatment of CVDs. However, small molecules that inhibit CARD9 based on the *CARD9*-protective variant (CARD9Δ11) have been identified for infectious bowel disease (IBD). These molecules, i.e., BRD5529, BRD4098, BRD4203, and BRD8991, can mimic the CARD9Δ11 protective actions in IBD to disrupt the direct interaction between CARD9 and TRIM62 and inhibit CARD9 ubiquitination and NF-κB activation 36. Meanwhile, the deubiquitinase USP15 was shown to remove TRIM62-mediated CARD9 ubiquitination and suppress CARD9-mediated signaling *in vitro* 102. Given the above, targeted CARD9 ubiquitination could be a potential therapeutic strategy for CVDs.

Due to the indispensable role of the CBM complex in the innate immune response, its disruption is another potential therapeutic strategy for CVDs. Zinc supplementation can prevent obesity-related cardiac hypertrophy in a mouse model by inhibiting CARD9/BCL10 signaling 45. Picomolar zinc can bind to the CARD domain of CARD9 to inhibit the polymerization of CARD9-CARD into helical assemblies and prevent BCL10 nucleation, thus suppressing the propagation of CARD9/BCL10 signaling 18. Therefore, zinc may be another form of CARD9 signaling inhibitor.

Other therapeutic strategies, such as the inhibition of BCL10 or MALT1, have been studied in the context of lymphocyte-mediated immune diseases 103, 104, and their applications in CVDs remain to be investigated.

## Conclusions and perspectives

In innate immunity, CARD9 signaling is involved in the transduction of signals from PRRs, including membrane-bound PRRs (Dectin-1, Dectin-2, and Mincle) and cytosolic PRRs (NOD2, RAD50, and RIG-1) in response to microbial infection. The pro-inflammatory effects induced by CARD9 signaling have been confirmed to involve cardiac injury and remodeling. Thus, the inhibition of CARD9 based on its role in inflammation is a promising strategy to treat CVDs. In contrast, CARD9-mediated anti-apoptosis and autophagy in cardiomyocytes showed protective effects on CVDs. Given the limited study on CARD9-mediated apoptosis and autophagy in CVDs, its exact role in different cell types (myeloid cells and cardiomyocytes) needs further exploration.

There are still some issues that require further attention on CARD9-mediated inflammation in CVDs. Firstly, the mechanisms underlying the regulation of CARD9 signaling in inflammation should be investigated, including: 1) The recognition of endogenous DAMPs, the main upstream molecules of CARD9 in various CVDs, and the crosstalk with other signaling molecules (like Toll-like receptors), should be elucidated to ensure normal immune responses against pathogens and prevent the overactivation of inflammatory responses following cardiac injury; 2) The epigenetic modifications (including phosphorylation and ubiquitination) and the CBM complex regulation of CARD9 should be taken account into the future study for developing potential CVDs treatment strategies. Secondly, the link between CARD9 and its family proteins, especially CARMA1, which is mainly expressed on lymphoid cells and forms the CBM complex for NF-κB activation, also needs attention for the better understanding the immune macroenvironment of certain CVDs.

## Figures and Tables

**Table 1:** CARD9 signaling in various CVDs.

Disease model	Name of Studied Gene	Expression Change of Studied gene	Immune Cells of Infiltration	Inflammatory Molecules	Signaling	Cardiac/Vascular Effects	Ref.
Viral Myocarditis	CARD9	not mentioned	not mentioned	IFN-γ, IL-6, TGF-β, IL-17A	not mentioned	CARD9 KO ameliorated myocardial inflammation and injury.	10
Cardiac Arteritis	CARD9	not mentioned	monocytes neutrophils	IL-1β CCL2/MCP-1, CXCL-1	Dectin2/CARD9/NLRP3	CARD9 KO mice were completely protected from CAWS-induced vasculitis.	39
Atherosclerosis	CARD9	not mentioned	macrophages	MCP-1	not mentioned	Hematopoietic CARD9 KO increased severity of plaque and lesion size.	46
Atherosclerosis	CARD9	not mentioned	macrophages CD3-positive cells	none	not mentioned	Hematopoietic CARD9 KO did not affect lesion size.	47
M-I/R Injury	CARD9	not mentioned	neutrophils	TNF-α, IL-6, CXCL-1, MCP-1	p38 MAPK	CARD9 KO decreased myocardial infarct size.	11
M-I/R Injury	CARD9	upregulation	NA	NA	CARD9/Rubicon	CARD9 KO impaired autophagy and increased cardiac dysfunction.	49
M-I/R Injury	CARD9	upregulation	NA	NA	CARD9/Apaf-1	CARD9 knockdown increased the apoptotic rate of H9C2 cells *in vitro* and aggravated cardiomyocytes apoptosis *in vivo*.	50
Ang II-induced Cardiac Injury	CARD9	upregulation	macrophages	IL-1β, TGF-β, CTGF	p38 MAPK NF-κB	CARD9 KO decreased cardiac inflammation and fibrosis.	12
Obesity-induced Cardiac Injury	CARD9	upregulation	macrophages	IL-6, TNF-a, IL-1β	p38 MAPK	CARD9 KO increased cardiac autophagy and decreased cardiac fibrosis and dysfunction.	44
Hypertension (Transverse Aortic Constriction-induced)	CARD9	not mentioned	not mentioned	not mentioned	NF-κB	CARD9 KO mice increased cardiac autophagy and did not develop cardiac fibrosis, hypertrophy, and dysfunction.	43

## Abbreviations

CVDs: cardiovascular diseases
CARD9: caspase recruitment domain-containing protein 9
PRRs: pattern recognition receptors
I/R: ischemia reperfusion
Ang II: angiotensin II
CARD: caspase recruitment domain
MAGUK: membrane-associated guanylate kinase
CCD: coiled-coil domain
BCL10: B-cell lymphoma/leukemia 10
PAMPs: pathogen-associated molecular patterns
CLRs: C-type lectin receptors
SYK: spleen tyrosine kinase
PKC: protein kinase C
MALT1: mucosa-associated lymphoid tissue 1
CBM: CARD9/BCL10/MALT1
NLRP3: NLR family pyrin domain-containing 3
NOD2: nucleotide-binding oligomerization domain-containing protein 2
RIG-1: retinoic acid inducible gene I
DAMPs: damage-associated molecular patterns
CVB3: coxsackievirus B3
KO: knockout
CAWS: *Candida albicans* water-soluble extract
MI: myocardial infarction
TAC: transverse aortic constriction
WT: wild-type
HFD: high fat diet
HF: heart failure
oxLDL: oxidized low-density lipoprotein
ICs: immune complexes
BM: bone marrow
BMDCs: bone marrow-derived dendritic cells
Mo-DCs: monocyte-derived dendritic cells
Apaf-1: apoptotic protease activating factor-1
H2O2: hydrogen peroxide
H/R: hypoxia/reoxygenation
UVRAG: UV-irradiation-resistance-associated gene
PI3KC3: phosphatidylinositol 3-kinase catalytic subunit type 3
Vps16: vacuolar protein sorting-associated protein 16 homolog
LAD: left anterior descending
IBD: infectious bowel disease
